# A global, cross cultural study examining the relationship between employee health risk status and work performance metrics

**DOI:** 10.1186/s40557-017-0172-1

**Published:** 2017-06-12

**Authors:** Ana Howarth, Jose Quesada, Peter R. Mills

**Affiliations:** 1 Cigna, Global Wellbeing Solutions Ltd, 24 Southwark Bridge Road, London, SE1 9HF UK; 2 Population Health Research Institute, St George’s, University of London, London, UK; 3 Cigna Health and Life Insurance Company, Bloomfield, CT USA; 4 Department of Respiratory Medicine, The Whittington Hospital NHS Trust, Highgate Hill, London, N19 5NF UK

**Keywords:** Employee well-being, Health risk assessment, Workforce productivity, Health risk profiling, Presenteeism, Absenteeism

## Abstract

**Background:**

Health risk assessments (HRA) are used by many organisations as a basis for developing relevant and targeted employee health and well-being interventions. However, many HRA’s have a western-centric focus and therefore it is unclear whether the results can be directly extrapolated to those from non-western countries. More information regarding the differences in the associations between country status and health risks is needed along with a more global perspective of employee health risk factors and well-being overall. Therefore we aimed to i) quantify and compare associations for a number of health risk factors based on country status, and then ii) explore which characteristics can aid better prediction of well-being levels and in turn workplace productivity globally.

**Methods:**

Online employee HRA data collected from 254 multi-national companies, for the years 2013 through 2016 was analysed (n = 117,274). Multiple linear regression models were fitted, adjusting for age and gender, to quantify associations between country status and health risk factors. Separate regression models were used to assess the prediction of well-being measures related to productivity.

**Results:**

On average, the developing countries were comprised of younger individuals with lower obesity rates and markedly higher job satisfaction compared to their developed country counterparts. However, they also reported higher levels of anxiety and depression, a greater number of health risks and lower job effectiveness. Assessment of key factors related to productivity found that region of residency was the biggest predictor of presenteeism and poor pain management was the biggest predictor of absenteeism.

**Conclusions:**

Clear differences in health risks exist between employees from developed and developing countries and these should be considered when addressing well-being and productivity in the global workforce.

**Electronic supplementary material:**

The online version of this article (doi:10.1186/s40557-017-0172-1) contains supplementary material, which is available to authorized users.

## Background

Occupational health risk assessment (HRA) developed out of the need to address poor work environments suffered by those most vulnerable, such as manual labourers and children [1]. Since then, employee HRA has been utilized on a global scale and has become fundamental to informing well-being programs [2] and employer strategies for increasing overall productivity [3]. What was initially considered an extra within large corporations, has become firmly entrenched within many organizations as not only a good investment strategy but as a duty of care. This is supported by a large body of evidence suggesting that well-being programs are effective in improving numerous aspects of employee health [2] with even the measurement of HRA alone potentially improving well-being levels [4]. Furthermore, research examining return on investment (ROI) [5, 6] has established links between well-being programmes that have been developed based on HRA, and increased productivity, mainly through the lowering of absenteeism and presenteeism [7–9].

HRA is the key to this relationship as it is the basis for understanding how health risk factors combine to drive absenteeism and presenteeism. Absenteeism is defined specifically as any time when an employee does not attend work, hence a clear cut loss of productivity. Presenteeism is more ambiguous as it is when an employee attends work but is not fully functionally due to illness, work overload or decreased motivation. Well-being programmes that focus on employee behaviour changes such as smoking cessation and physical activity improvement have shown biological improvements, such as reduced blood pressure and BMI (body mass index) [10–12], as well as improvements in absenteeism and presenteeism. Further research has provided evidence that psychosocial markers such as increased job satisfaction and lowered stress levels can also be improved by well-being programmes [13]. Importantly, many of these changes have also been linked to reduced medical costs [14–16].

There are many potential drivers for organizations to implement HRA and well-being programs, ranging from reducing costs to delivering on a duty of care, but whatever the underlying reason it is clear that the workplace is an ideal setting for health promotion as it facilitates access to large populations. In fact, the World Health Organization (WHO) Programme for Occupational Health recently advocated the workplace as a priority for health promotion programs for both public and private organizations so as to achieve success in the globalizing marketplace [17]. A review of the literature produces various examples of large companies reporting substantial savings in medical costs equaling far more than they spent on well-being programs [18]. However, one obstacle with HRA, and therefore well-being programmes, is the current western-centric focus of HRAs. Occupational well-being programmes are predominately utilized by and developed for North American and European populations [2, 5, 12, 19, 20]. As a consequence, specific health risk profiles from non-western or developing countries have seldom been compared and the few times they have, significant differences have been found across many measures [21]. As there is much less known about employee health risk factors relevant to developing countries it is unlikely HRAs are being adapted to local conditions effectively. In fact, evidence suggests that many programmes that have been implemented include only minor adjustments such as measurement conversion (e.g. metric to imperial units) [22].

### Aims

In an attempt to fill gaps in the literature regarding developing versus developed country health risk factors, we used HRA data collected from 254 multi-national companies across the globe representing numerous industries, but including consumer goods, pharmaceutical, finance and telecommunications, from the years 2013 to 2016. This data was based on a previously validated HRA [23] designed specifically for corporate employee populations and which was translated into 28 languages and localized for different populations. Our first aim was to investigate if there were health risk differences between developed and developing countries. Secondly, we aimed to explore factors that predict higher well-being levels and subsequently better productivity. Overall, it is hoped that a more global view of the state of health risk assessment and wellness can be offered.

## Methods

### Study design and population

This study used a cross-sectional design to summarize and compare findings from online HRA’s completed by multi-national organizations between the years 2013 to 2016. The HRA was administered as an online questionnaire to 254 companies from 120 countries. All employees received an email with a link and a password to log in to the survey. Two reminders were sent out after the first email to encourage engagement. The questionnaire was based on the previously validated HRA which was developed to generate data specific enough to inform well-being interventions within occupational settings [23]. It consisted of self-report questions including demographics, health risk factors, basic health screenings and lifestyle behaviours. A sample of the original questionnaire can be seen in Additional file 1.

### Study sample

The final sample consisted of 117,274 employees (aged 18 to 64 years) taken over four years from the period 2013 to 2016.

### Measures

#### Key variables

To assess presenteeism and absenteeism, self-reported job effectiveness and medical score ratings (i.e. a combination of sick days taken and number of illness conditions) were use as respective proxy outcome measures.

#### Job effectiveness score

Job effectiveness was a score derived from the combination of two items from the online questionnaire: “How effective have you been over the last 3 months” based on the original validated questionnaire [23] and “How much do you think your overall health has impaired your work performance over the last 3 months?”. As presenteeism can be challenging to measure, the most common approach is to query how much an employee believes their work is effected by their health. The job effectiveness score covers this efficiently.

#### Medical score

Medical score was the score derived from the combination of two items from the online questionnaire: “Do you have any of the following conditions?” in relation to a checklist of 18 possible illness conditions and “During the past 3 months, how much time have you missed from work due to illness or injury?”. The combination of sick days taken and presence of medical conditions, which increases possibility of future sick days was considered an appropriate proxy for employee absenteeism for analyses. The list of possible medical conditions can be seen in Additional file 2. For both job effectiveness and medical health scores, higher scores represent better functioning.

#### Country status

Although a debate exists within the literature regarding classification of countries and level of development, for the purposes of this paper, a classification system reported in a United Nations, World Economic Situation and Prospects report [24] was used as the basis for classifying countries as either developed or developing. Any countries that are considered in transition were included with the developing countries group for simplification.

#### Other variables

Other variables included were derived scores representing jobs satisfaction, physical activity, pain, general perception of health, sleep, mood, nutrition and stress. These scores were comprised of one or more items from the online questionnaire, combined and then weighted accordingly to make a standardised score on a scale from 0 to 100 (with 0 being the poorest score and 100 being the best possible).

Age and gender were included so that they could be controlled for but other socio-demographic information such as region of residency, number of dependents, marital status, BMI and heart risk factors were also collected so as to better describe the sample. Finally, health risk factors as an outcome measure was also included and this was a simple summation of the number of health risk factors self-reported. Examples of health risk factors include, smoking, existing medical illnesses, or presence of obesity.

### Statistical methods

Descriptive statistics were used to summarise the data for each country status group. Multiple linear regression was used to examine the associations between country status and health outcome variables.

In order to build predictive models for both presenteeism and absenteeism, a forwards stepwise approach was used with each used as an outcome variable. A number of predictors were used to try and build the model which, using the available data, could best predict each of our outcomes of interest. The most highly statistically significant variables were included first and the predictive importance of further variables were assessed using the differences in the R-squared values from models including and excluding the variable of interest. The difference in this statistic indicated the predictive importance of the parameter being tested and a threshold of inclusion was set such that a predictor was required to have an importance level of 0.01 or higher.

Finally once all possible predictors had been tested, we were left with a model containing all predictors with an importance level greater than or equal to 0.01. All analyses were done using SPSS v24 for Windows with the level of significance set at *p* < 0.05.

## Results

### Descriptive statistics

The analysis sample consisted of 117,274 individuals; 30,104 from developing countries and 87,170 from developed countries. Sample characteristics are presented in Table 1. A similar proportion of people were observed for most variables of interest across country status with the exception of age, where those from the developing world were on average approximately 7 years younger. Furthermore, the developing countries were made up of 88 countries compared to only 32 from their developed counterparts. The developing country data comprised largely of individuals working in India (*n* = 10,154, 33.7%) whereas the majority of the developed country data was from Canada (n = 23,841, 27.4%). Further breakdown of size of countries can be found in Additional file 3.

**Table 1:** Sample characteristics (*N* = 117,274)

Variable^a^	Developing *N* = 30,104	Developed *N* = 87,170
Number of countries, N	88	32
Age (years), Mean, (SD)	33.3 (9.0)	40.5 (10.6)
Male	60.7	59.0
Marital Status (Married/partner)	57.6	69.4
Have dependents	45.8	52.3
BMI obese^b^	15.2	24.3
Heart disease risk factors^c^	15.1	14.7
Anxiety or depression	18.8	14.2
One day or less sick days taken	85.3	86.2
One or less health risk factors	17.9	27.3
Size of countries		
< 10	40	0
10–100	21	8
101–1000	18	15
1001–10,000	7	6
10,001–25,000	1	3

^a^ % unless otherwise stated

^b^ BMI (Body mass index, kg/m2) score of ≥30

^c^ Have high blood pressure and/or high cholesterol

Means and standard deviations of the HRA scores from developing and developed countries for key variable are presented in Table 2. Crude averages appear to suggest an overall better self reported health status in developed countries compared to the developing. However, it also appears that developed countries have lower level of job satisfaction and medical scores.

**Table 2:** Health risk assessment scores (Mean, SD)

Health Outcome Variables^a^	Developing *N* = 30,104	Developed *N* = 87,170
	Mean (SD)	Mean (SD)
Activity	51.2 (25.6)	54.1 (29.0)
Job effectiveness	63.6 (23.5)	73.2 (22.3)
Job satisfaction	74.2 (21.8)	70.9 (24.3)
Medical	65.4 (32.3)	62.5 (33.0)
Nutrition	31.6 (16.8)	41.33 (22.3)
Perception of general health	56.4 (19.0)	60.8 (19.7)
Stress	44.9 (18.1)	49.3 (19.8)
Overall well-being score	45.8 (18.9)	47.3 (20.1)

^a^ All scores on a scale from 0 (poorest) to 100 (best possible)

#### Developing versus developed countries

Multiple linear regression results (Additional file 4) showed developed countries to have significantly higher scores across all variables of interest with the exception of one. After adjusting for age and gender, on average, respondents from developed countries had an activity score of 6.25 points higher, job effectiveness score of 8.35 points higher, medical score of 1.17 points higher, nutrition score of 7.96 points higher, perception of general health score of 3.80 points higher, stress score of 2.94 points higher and an overall score of 3.30 points higher than developing countries. The exception to this was job satisfaction, which had a score of 4.14 points lower.

### Prediction of productivity

#### Presenteeism

**Fig. 1:**
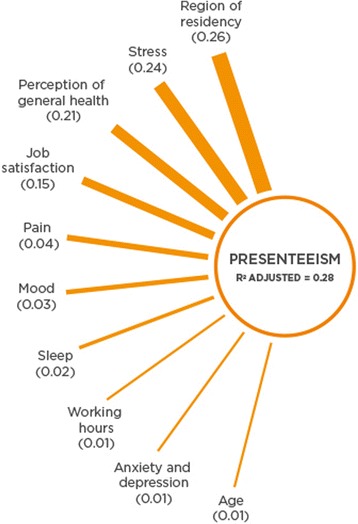
Presenteeism predictors in order of importance from top to bottom according to predictor importance

Figure 1 is a diagram illustrating the most important predictors of presenteeism. The biggest predictors (with an importance level greater than 0.01) of job effectiveness in descending order were: region of residency (0.26), stress (0.24), perception of general health (0.21), job satisfaction (0.15), pain (0.04), mood (0.03), sleep (0.02), working hours (0.01), anxiety and/or depression (0.01) and lastly age (0.01).

In Table 3, predictor variables with coefficients, confidence intervals, *p* values and predictor importance are reported for the outcome variable of job effectiveness. From this model it appears that some regions of residency (i.e. Europe and North American) were related to decreases in presenteeism while others (i.e. Asia and Latin American) were related to increases. Increases in scores for stress, job satisfaction, pain, mood, sleep (all of which higher scores indicate better management or less negative outcomes) and age predicted decreases in presenteeism. Decreased working hours predicted increases in presenteeism with working less than 30 h per week having the highest impact and working 41–50 h per week having the lowest impact on presenteeism increase. The absence of anxiety or depression predicted lower presenteeism as well.

**Table 3:** Prediction model for presenteeism including variables with an importance level ≥ 0.01

Predictor Variables	Presenteeism		
	*Coefficient* (95% CI)	Predictor Importance	*P*
Region			
Region 2 (Asia)	−7.47 (−8.06, −6.88)	0.26	<0.001
Region 3 (Europe)	0.76 (0.22, 1.30)	0.26	< 0.001
Region 4 (Latin America)	−1.85 (−2.51, −1.19)	0.26	< 0.001
Region 5 (North America)	2.71 (2.17, 3.24)	0.26	< 0.001
Stress	0.26 (0.25, 0.27)	0.24	< 0.001
Perception of general health	0.19 (0.18, 0.19)	0.21	< 0.001
Job satisfaction	0.11 (0.11, 0.12)	0.15	< 0.001
Pain	0.04 (0.04, 0.05)	0.04	< 0.001
Mood	0.08 (0.07, 0.09)	0.03	< 0.001
Sleep	0.05 (0.05, 0.06)	0.02	< 0.001
Working hours			
Less than 30 h/week	−4.52 (−5.42, −3.63)	0.01	< 0.001
30–40 h/week	−1.42 (−2.02, −0.82)	0.01	< 0.001
41–50 h/week	−0.68 (−1.23, −0.12)	0.01	0.02
51–60 h/week	−1.36 (−1.96, −0.77)	0.01	< 0.001
No anxiety and/or depression	1.98 (1.64, 2.31)	0.01	< 0.001
Age	0.06 (0.05, 0.07)	0.01	< 0.001

### Absenteeism

**Fig. 2:**
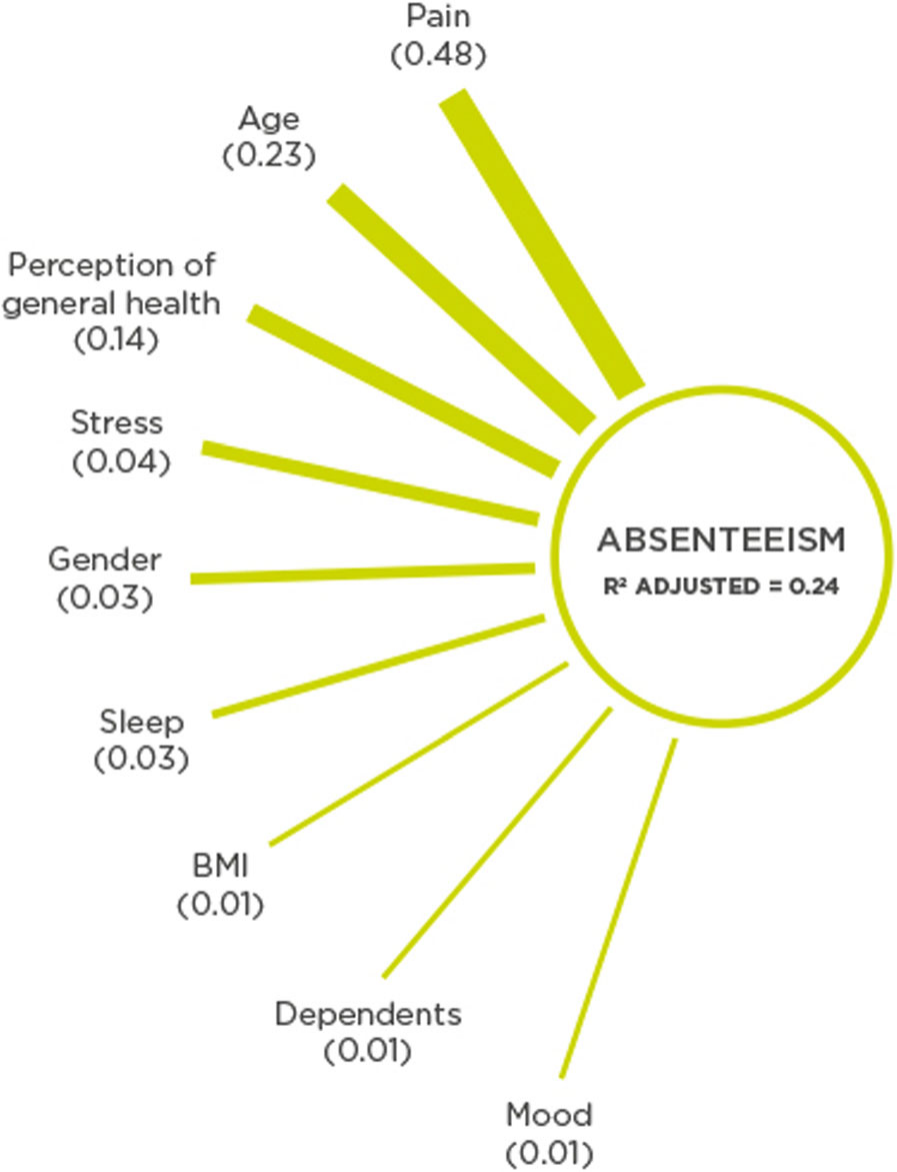
Absenteeism predictors in order of importance from top to bottom according to predictor importance

Figure 2 is a diagram illustrating the most important predictors of absenteeism. The biggest predictors (with an importance level greater than 0.01) of lower absenteeism in descending order were: pain (0.48), age (0.23), perception of general health (0.14), stress (0.04), gender (0.03), sleep (0.03), BMI (0.01), having dependents (0.01), and lastly mood (0.01).

In Table 4, predictor variables with coefficients, confidence intervals, predictor importance and *p* values are reported for absenteeism. Increases in scores for pain, perception of health, stress, and mood (where higher scores means better management and better perceptions respectively) predict lower absenteeism. Conversely, increases in age, BMI and being female, predicts higher absenteeism.

**Table 4:** Prediction model for absenteeism including variables with an importance level ≥ 0.01

Predictor Variables	Absenteeism		
	*Coefficient* (95% CI)	Predictor Importance	*p*
Pain	0.25 (0.25, 0.26)	0.48	< 0.001
Age	−0.57 (−0.59, −0.55)	0.23	< 0.001
Perception of general health	0.26 (0.25, 0.27)	0.14	< 0.001
Stress	0.16 (0.15, 0.17)	0.04	< 0.001
Sleep	0.08 (0.07, 0.09)	0.03	< 0.001
Gender (female)	−4.46 (−4.82,−4.09)	0.03	< 0.001
BMI	−0.27 (−0.31, −0.23)	0.01	< 0.001
Dependents (1 or more children)	2.55 (2.17, 2.93)	0.01	< 0.001
Mood	0.07 (0.06, 0.08)	0.01	< 0.001

## Discussion

Overall, comparisons based on country status (i.e. those classified as developing or developed), demonstrated that most health risks were slightly more prevalent for populations from developing countries. This mirrors previous research [21] and is potentially explained by the fact that many developing countries have less resources and access to healthcare [25]. In addition, it was found that individuals from the developing countries had a higher number of risk factors on average. Only 17.9% of individuals from developing countries reported having only one health risk factor (e.g. smoking or existing medical illness) or less while more than a quarter of the developed countries group (27.3%) reported this status. These findings are particularly valuable to industry as heath risk factors highlighted by HRA, have been repeatedly related to productivity and are highly modifiable with the support of effective well-being programs.

One area where the developing group was better than the developed group was obesity, as only a sixth (15.2%) of the developing group were classified as obese (i.e. BMI of 30 or over), and almost a quarter of the developed group (24.3%) was obese. While this represents a current advantage, there is a growing trend of lifestyle related illness in developing countries, that once were considered the sole remit of western nations. Anxiety, depression and heart disease risk factors, such as high blood pressure and cholesterol, are now spreading to developing countries where there were once far lower rates [26] so differences in obesity may disappear with time. In fact, in this study the developing country group reported higher rates of anxiety and/or depression (18.8%) than in developed countries (14%) as well as slightly higher heart risk factors.

As the developing group was younger by approximately seven years, this paints a picture of a young, highly anxious and perhaps very stressed employee group. However, when reviewing the standardised scores, job satisfaction was slightly higher in the developing group. It is hard to be certain why, considering developed countries with higher income may afford more agreeable work environments but it may be a reflection of the value of employment in countries where the unemployment rates are high and there is a great socioeconomic consequence of being unemployed. As well, employees in this study belonged to large, multi-national corporations for which employment may be valued even more in developing countries.

When associations between country status and health outcome factors were examined, while controlling for age and gender, all standardised scores (e.g. activity, perception of general health, stress and nutrition) were higher in developed countries, which makes intuitive sense considering the associated wealth and level of available healthcare resources. Gender and age influenced differences in some circumstances, with lower levels of nutrition being associated with men and medical scores decreasing with age, but overall, these findings and most associations reflected previous socioeconomic models of global health inequalities [25].

Analysis of the sample as a whole in relation to predicting well-being measures and subsequent productivity, resulted in a variety of predictive health factors for absenteeism and preseenteeism. Some of these factors were shared but the combination of strongest predictors for each were unique. Factors that predicted lower presenteeism included region of residency, stress and employee perception of general health. Region of residency was used in this analysis instead of country status so as to tease out more cross-cultural differences. Regions predominately consisting of developed countries (i.e. Europe and North America) appeared to have an advantage and predicted lower presenteeism. Lifestyle factors such as better stress, mood and sleep management, along with job satisfaction, were also related to lower presenteeism These factors all support previous occupational research in relation to presenteeism [27–31].

Conversely, predicting absenteeism was slightly more complex as reported absenteeism rates were extremely low. This may be a reflection of the employees being less likely or able to take leave of absence when ill, across both higher and lower income countries. Interestingly, the strongest predictor for lower absenteeism overall was good pain management which in light of the widespread issue of chronic pain is perhaps a good indication of how crucial relevant HRAs can be. Following this, lifestyle factors such as good stress management, positive mood and perception of general health and younger age, all predicted lower absenteeism. Factors which predicted higher absenteeism included BMI, being female (which has long been well documented and was therefore controlled for) and having one child or more, which is in direct contrast to previous research so possibly worth examining more closely in future.

Overall, most factors that were found to drive both presenteeism and absenteeism support previous occupation productivity and well-being research [15, 22, 23] with the exception of global region, which has not previously been explored in much depth.

### Limitations

While the sample size and breadth of countries included were substantial, some conclusions may still be ambiguous due to the limitations of the data available. Developing country data included employees that were not necessarily representative of the general working populations of these countries by virtue of the fact that they were employed by multi-national corporations. This means interpretation of results must remain conservative.

Furthermore, although the HRA scores were based on the questionnaire that was adapted for use in various countries, it was still originally designed for use in western-centric countries making it difficult to ascertain how relevant it was in measuring non-western-centric populations. Well-being measures and health risk profiles are still under-represented in many of the countries that were included and it is important to remember that the proportion of developing countries included was only one third of the size of the developed countries group. This is compounded by the fact that both groups favoured one country in representation substantially more than others, again, limiting the scope for a truly global perspective.

Finally, while the HRA was very robust for the purpose and population it was developed for, data produced was unwieldy at times when used for comparison with other studies as HRA assessment varies so much across the literature and there is a lack of standardized instruments being currently used.

## Conclusions

As this study was able to draw upon a substantial sample involving 120 countries across a variety of health measures, the findings were both supportive of previous research and insightful in areas not yet explored globally. Associations between health status and productivity are extensive. However, despite previously mentioned limitations, it is clear that there are differences in the type and number of heath risk factors between developing and developed countries. This accentuates the importance of using population specific HRA across diverse work place settings as the development of effective well-being programmes is dependent on targeting relevant health risk factors.

Finally, the results related to HRA in general are very beneficial to corporations as it is clear that health risk factors related to increasing productivity are highly modifiable with well-being programs globally.

## Additional files

Additional file 1: Health & Well-being (HWB) Questionnaire, sample of online questionnaire. (DOCX 24 kb)

Additional file 2: Medical conditions. List of medical conditions included in online HRA. (DOCX 10 kb)

Additional file 3: Breakdown of countries and respondent numbers. Supplementary table of country breakdown according to number of respondents and country status. (DOCX 20 kb)

Additional file 4: Table S1. Associations between country status and health outcome variables. (DOCX 16 kb)

## Abbreviations

BMI: Body mass index
CI: Confidence interval
HRA: Health risk assessment
ROI: Return on Investment
SD: Standard deviation
SPSS: Statistical package for the social science
US: United States of America
WHO: World Health Organisation
