# Supplementary material for: A global, cross cultural study examining the relationship between employee health risk status and work performance metrics
Source: Ann Occup Environ Med. 2017 Jun 12;29:17. doi: 10.1186/s40557-017-0172-1 (PMC5469053; doi:10.1186/s40557-017-0172-1)
Supplement: Supplementary file 2 — Medical conditions. List of medical conditions included in online HRA. (DOCX 10 kb) [file 40557_2017_172_MOESM2_ESM.docx]

**Additional file 2: List of medical conditions included in HRA**

Do you have any of the following conditions?

Mark all that apply.

Anxiety

Arthritis

Asthma

Back, neck or spinal problems

Bronchitis or emphysema (often called COPD)

Cancer

Depression

Diabetes

Eczema or other skin condition

Hayfever or similar allergy

Heart disease (heart attack, angina, angioplasty, bypass surgery or heart failure)

Heartburn or acid reflux (often called GERD)

High cholesterol

High blood pressure (often called hypertension)

Migraine headaches

Osteoporosis

Stroke

Stomach ulcer (gastric or duodenal ulcer)

Any other serious health problem for which you are receiving medical treatment
