# Supplementary material for: A global, cross cultural study examining the relationship between employee health risk status and work performance metrics
Source: Ann Occup Environ Med. 2017 Jun 12;29:17. doi: 10.1186/s40557-017-0172-1 (PMC5469053; doi:10.1186/s40557-017-0172-1)
Supplement: Supplementary file 3 — Breakdown of countries and respondent numbers. Supplementary table of country breakdown according to number of respondents and country status. (DOCX 20 kb) [file 40557_2017_172_MOESM3_ESM.docx]

Breakdown of countries and respondent numbers

Addition file 3. Country breakdown according to number of respondents and country status

| **Developing Countries N= 88** | | | | | | | **Employee No.** |
| --- | --- | --- | --- | --- | --- | --- | --- |
| Algeria  Bahamas  Barbados  Bolivia  Brunei  Cayman Islands  Cyprus  Ethiopia  Jordan  Mali | | Malta  Niger  Reunion  Saint Kitts and Nevis  Rwanda  Uganda  Uzbekistan  Zambia  Bosnia and Herzegovina  Chad | | Democratic Republic of the Congo  Georgia  Kazakhstan  Kyrgyzstan  Libya  Papau New Guinea  Sudan  Mozambique  Kenya  Kosovo | Gabon  Lebanon  Macau  Sierra Leone  Slovenia  Tanzania  Turkmenistan  Bahrain  Cameroon  Cote D’Ivoire | | < 10 |
| AOman  Trinidad and Tobago  Kuwait  Egypt  Qatar | | Costa Rica  Iraq  Serbia and Montenegro  El Salvador  Honduras | | Israel  Nicaragua  Uruguay  Angola  Taiwan | Dominican Republic  Morocco  Bangladesh  Saudi Arabia  Korea  Equatorial Guinea | | 10-100 |
| Peru  Afghanistan  Turkey  Singapore | | Chile  Russia  Vietnam  United Arab Emirates | | Hong Kong  Nigeria  Indonesia  Guatemala  Argentina | Venezuela  Panama  Colombia  Ecuador  South Africa | | 101-1000 |
| Malaysia | | Thailand  Philippines | | India  China | Mexico  Brazil | | 1001-15,000 |
| **Developed Countries N= 32** | | | | | |  | **Employee No.** |
| Latvia | Lithuania | | Croatia  Luxemburg | | Greece  Austria | Hungary  Estonia | 10-100 |
| Slovakia  Switzerland  New Zealand | Japan  Romania  Czech | | Bulgaria  Italy  Norway | | Portugal  Denmark  Spain | France  Poland  Germany | 101-1000 |
| Belgium | Finland | | Netherlands | | Ireland | Sweden  Australia | 1001-10,000 |
| United Kingdom | United States of America | | | | Canada | | 10,001-25,000 |
