# Supplementary material for: A global, cross cultural study examining the relationship between employee health risk status and work performance metrics
Source: Ann Occup Environ Med. 2017 Jun 12;29:17. doi: 10.1186/s40557-017-0172-1 (PMC5469053; doi:10.1186/s40557-017-0172-1)
Supplement: Supplementary file 4 — Associations between country status and health outcome variables. (DOCX 16 kb) [file 40557_2017_172_MOESM4_ESM.docx]

**Additional file 4: Table S3 Associations between country status and health outcome variables**

**Unstandardized coefficient*

|  | **Outcome Variables**  **N= 117, 274** | | | | | | | |
| --- | --- | --- | --- | --- | --- | --- | --- | --- |
| **Predictor Variables** | **Activity** | **Job Effectiveness** | **Job Satisfaction** | **Medical** | **Nutrition** | **Perception of General Health** | **Stress** | **Overall HWB** |
|  | ***B** (95% CI)** | ***B* (95% CI)** | ***B* (95% CI)** | ***B* (95% CI)** | ***B* (95% CI)** | ***B* (95% CI)** | ***B* (95% CI)** | ***B* (95% CI)** |
| **Country Status** |  |  |  |  |  |  |  |  |
| Developed | 6.25  (5.87, 6.63) | 8.35  (8.04, 8.66), | -4.14  (-4.46, -3.81) | 1.17  (0.72, 1.61) | 7.96  (7.68, 8.25) | 3.80  (3.54, 4.07) | 2.94  (2.68, 3.21) | 3.30  (3.03, 3.57) |
| Developing | [Baseline group] | | | | | | | |
| **Age** | -0.44  (-0.45, -0.42) | 0.18  (0.17, 0.20) | 0.12  (0.11, 0.14) | -0.55  (-0.57, -0.53) | 0.23  (0.22, 0.25) | 0.08  (0.07, 0.09) | 0.21  (0.20, 0.22) | -0.25  (-0.26, -0.23) |
| **Gender** |  |  |  |  |  |  |  |  |
| Male | 7.90  (7.58, 8.23) | 1.64  (1.38, 1.90) | 0.67  (0.40, 0.95) | 8.26  (7.88-8.63) | -4.98  (-5.22, -4.74) | 1.23  (0.07, 0.10) | 5.45  (5.23, 5.67) | 4.00  (3.72, 4.18) |
| Female | [Baseline group] | | | | | | | |
